# Born to win? Investigating the relative age effects in the big five European women's football leagues

**DOI:** 10.3389/fspor.2025.1546913

**Published:** 2025-04-11

**Authors:** Benito Pérez-González, Iyán Iván-Baragaño, José Bonal, Jairo León-Quismondo, Álvaro Fernández-Luna, Pablo Burillo

**Affiliations:** ^1^ Facultad de Economía y Empresa, Universidad Internacional de la Rioja, Madrid, Spain; ^2^ Department of Sports Sciences, Faculty of Medicine, Health and Sports, Universidad Europea de Madrid, Villaviciosa de Odón, Spain; ^3^ Real Madrid Graduate School, Faculty of Medicine, Health and Sports, Universidad Europea de Madrid, Villaviciosa de Odón, Spain

**Keywords:** relative age effects, soccer, talent identification, birth date, development, maturation

## Abstract

**Introduction:**

This study examines the presence of the Relative Age Effects (RAEs) among players in the top five European women's football leagues during the 2023/24 season.

**Methods:**

A total of 1,634 professional players from the Women's Super League (England), Liga F (Spain), Frauen-Bundesliga (Germany), Serie A Femminile (Italy), and Division 1 Féminine (France) were analyzed. Birth date distributions were assessed to determine the prevalence of RAEs both collectively and within each league.

**Results:**

Poisson regression analyses revealed significant overall RAEs, with a higher proportion of players born in the first semester of the year. Individually, significant RAEs were found in England, Spain, Italy, and France, while Germany did not exhibit statistically significant effects. When analyzed by playing position, significant RAEs were observed among goalkeepers and midfielders, but not among defenders and forwards.

**Discussion:**

These findings highlight the ongoing influence of RAEs in elite women's football and underscore the need for strategies to mitigate its impact on talent identification and player development.

## 1 Introduction

In recent years, women's football has experienced significant growth, particularly in European countries. Between 2019 and 2023, the number of registered female players increased by 45.03% in England, 66.7% in Spain, and 17.36% in France (1, 2). However, the United States still represents approximately 40% of all female football federation registrations worldwide (2), a statistic that justifies this nation's performance globally in the history of this sport.

This growth in participation has been accompanied by a notable increase in the volume of scientific research dedicated to women's football (3). This emerging volume of scientific research enabled researchers to transition away from the previously predominant unisex approach, wherein findings from men's football were indiscriminately applied to women's football due to the lack of sex-specific evidence (4). Among the underexplored topics in women's football research are the relative age effects (RAEs), a phenomenon extensively studied in men's football but less comprehensively examined in the context of the female game (5, 6).

The relative age effects in sports, particularly in football, is defined as the consequences on performance stemming from differences between athletes' chronological and biological ages (7, 8). In recent years, the understanding of this issue has led to establishing a series of conclusions regarding talent selection at early ages (9). Consequently, different approaches have been proposed to eliminate or reduce the influence of these effects, one of the most notable being bio-banding. This method consists of categorizing young athletes, typically aged 11–15, into groups or “bands” according to their estimated biological maturity rather than solely their chronological age for specific competitions and training sessions (10). The assessment of maturity is generally based on the predicted percentage of an individual's final height at a given point in time. However, the authors emphasize that bio-banding should be applied in specific contexts and used primarily for short-term purposes, such as training periods or experimental tournaments.

Another emerging concept in the literature on relative age effects in sports is the “underdog” phenomenon (11, 12) which has been explored as a hypothesis in other studies (13, 14). This theory posits that younger athletes within a given cohort face challenges from their relatively older peers. It suggests a reversal of the traditional RAEs perspective, arguing that what truly defines elite players is their ability to overcome disadvantages, including those imposed by RAEs. To support this claim, studies not only examine the presence of the effects but also track athletes' development and career trajectories from their early years. Finally, other models have also been considered in the literature to analyze relative age, such as the social agents theory (15). This approach examines how parents, coaches, and athletes themselves influence the effects of relative age. Similarly, the developmental systems theory has been explored, highlighting the constraints associated with RAEs (16). These constraints fall into three categories: individual (date of birth), task-related (type of sport), and environmental (social factors and sports development).

In the specific context of women's football, several authors have investigated the influence of these effects, although the results found are often contradictory (17–33). One of the first studies (25) on women's football was conducted in the United States and aimed to demonstrate the occurrence of this selection bias in players from the US Olympic Development Program. These authors showed a slight bias toward overrepresentation of players born in the first half of the year compared to the second. However, this effect was not observed when the analysis was conducted based on the quarter of birth (25).

Following this initial evidence, research in Asia has provided further evidence into the prevalence of RAEs. Matsuda and Ishigaki (19) observed, after analyzing a sample of over 4,000 players, that the percentage of players born in the first quarter of the year was approximately 10% higher compared to those born in the last quarter of the year. Nakata and Sakamoto (27) further examined sex differences in RAEs among Japanese athletes, highlighting distinct patterns between male and female players. In China, Li et al. (28) explored the impact of the “one-child” policy on elite soccer players, suggesting that structural constraints in talent selection may have influenced the observed RAEs.

In Europe, multiple studies have confirmed the presence of RAEs across different levels of competition, particularly among players from national and regional teams (22). Research in Switzerland (29) and other recent studies on elite female soccer players in international tournaments (30, 31) have further highlighted the prevalence of RAEs, especially in younger age categories, with positional and regional variations. Additionally, Delorme et al. (17) found that RAEs were evident in lower divisions of French football. However, relative age effects did not occur in adult players; on the contrary, there was an underrepresentation of players born in the first quarter of the year (17), which could be justified by the “underdog” theory (11), according to which future performance and the duration of sports careers could be greater for athletes born in the later quarters of the year. A broader analysis in Italy with a sample of 1,535 female basketball, volleyball, and football players, with greater effects observed in football (34). It is also interesting to note the study conducted in Germany, which analyzes RAEs from two perspectives: as within-year effects (WYEs), examined within the same birth year, and as between-year effects (BYEs), where athletes are grouped into two-year age bands. In the latter case, disadvantages are more pronounced (33).

On the other hand, numerous studies have not found evidence of relative age effects in women's football. For example, a recent study (21) conducted on the FIFA Women's World Cup championships held since 2007 in the absolute, U20, and U17 categories observed that relative age effects in the U17 and U20 samples were not significant until the years 2016 and 2018, respectively, when the effects were observed. Furthermore, no effects were observed in 2007, 2011, 2015, and 2019 editions of the FIFA Women's World Cup (21). Similarly, a recent investigation found no effects of relative age in 2,387 female players from the qualification squads/teams for the most recent European Championship campaigns (35).

In addition, other studies have also failed to detect significant RAEs in elite women's football. For instance, Riveiro et al. (31) analyzed under-17, under-20, and adult elite female soccer players, reporting no significant relative age effects in adult categories. Likewise, Delorme et al. (17) investigated the prevalence of RAEs in elite sports in France, considering the possible influence of gender. Their results indicated that, although RAEs are evident in men's sports, its presence in women's sport is less consistent, suggesting that the effects may vary depending on gender and the specific sport. Nakata and Sakamoto (27) examined RAEs among elite Japanese athletes and found that, in the case of female athletes, only volleyball showed significant RAEs. In other female sports analyzed, such as football, no skewed distribution of birthdates was observed. These findings highlight the complexity of relative age effects in women's football and suggest that its presence may be influenced by multiple contextual and structural factors.

Considering all the studies mentioned, it is essential to continue exploring the relative age effects in women's sports, specifically in the world of football. Therefore, this research aims to address two objectives: first, to determine the presence of relative age effects across the five major European leagues, both collectively and individually; and second, to evaluate whether these effects varies based on players' positional roles.

## 2 Materials and methods

### 2.1 Study design

This research employed a cross-sectional observational design to evaluate the presence of Relative Age Effects (RAEs) among players in the top five European women's football leagues (based on their economic impact, competitiveness, and visibility, as highlighted annually in Deloitte's Football Money League reports) during the 2023/24 season: the Women's Super League (England), Liga F (Spain), Frauen-Bundesliga (Germany), Serie A Femminile (Italy), and Division 1 Féminine (France). The study focused on the entire roster of registered players for each team within these leagues, aiming to provide a comprehensive view of RAEs at the elite level of European women's football.

### 2.2 Participants

The study sample consisted of 1,634 professional female football players distributed across the five leagues. To ensure accuracy, data collection was conducted between February 1 and February 10, 2024, shortly after the closure of the mid-season transfer window. Only players officially registered with their respective clubs at this time were included. Any players on long-term leave, such as maternity or injuries, were also considered part of the roster if they remained registered.

### 2.3 Data collection

Data were sourced from publicly available records on official club websites and the specialized football statistics platform Livefutbol (36). Information for each player included: full name, date of birth (used to determine the player's relative age within the competition year), playing position (classified as goalkeeper, defender, midfielder, or forward), and club affiliation.

Additional manual verification was performed to cross-check data discrepancies between club websites and the Livefutbol platform. This ensured consistency and accuracy of the dataset used in the analysis.

### 2.4 Determination of relative Age effects and data analysis

The standard cut-off date for categorization by birth year is January 1st. However, England's Women's Super League uses a September 1st cut-off date, in line with the academic year. For the English league, player birth dates were adjusted to align with this variation. Adjusted dates were used in subsequent calculations to maintain consistency across leagues.

Relative Age Effects (RAEs) were first evaluated and proved (*χ*^2^ = 46.77; *p* < 0.001) using the Chi-square goodness-of-fit test for a hypothetical equal distribution across the four quartiles. Similarly, an association analysis was conducted between the birth quartile variable and the league and position variables, taking into account that for this second analysis, the expected values for each of the positions and leagues were those observed in the overall sample (i.e., 27.4%, 28.4%, 22.2%, and 20.0%). The effect size for this association quantified using Cramer's V. Finally, Relative Age Effects were calculated through Poisson regression (37, 38). The Poisson regression formula *y* = *e* (b0 + b1x) serves to explain the frequency count of an event (y) by an explanatory variable x. The data used for Poisson regression were week of birth (WB) whereby the first week in January was designated WB 1, and time period of birth (Tb) describing how far from the beginning of the year a player was born. This last index ranging between 0 and 1 was calculated as Tb = (WB − 0.5)/52. In the Poisson regression, the event (y) was the frequency of birth in a given week and the explanatory variable (x) was Tb. We also calculated the index of discrimination (ID) according to Doyle and Bottomley (38) as e-b1. This index measures the relative odds of a player born on day 1 vs. day 365 of the competition year being selected. The likelihood ratio D^2^ was determined according to Cohen et al. (39). All statistical tests, including descriptive analysis, were performed using the software package R (version 4.3.2). Significance was set at *p* < 0.05.

## 3 Results

### 3.1 Distribution of birth dates by league

The distribution of players' birth dates according to their quartile (Q) of birth across the five leagues is summarized in Table 1.

**Table 1:** Association between birth quartile and league.

League	Q1 (*n* = 441–27.4%)	Q2 (*n* = 457–28.4%)	Q3 (*n* = 358–22.2%)	Q4 (*n* = 354–22.0%)	*P* [ES]
Division 1 Feminine	93 (28.8%)	92 (28.5%)	78 (24.1%)	60 (18.6%)	*p* = .33
FA Women's Super League	86 (29.0%)	77 (25.9%)	67 (22.6%)	67 (22.6%)	
Frauen – Bundesliga	79 (24.3%)	92 (28.3%)	77 (23.7%)	77 (23.7%)	
LigaF	100 (25.8%)	125 (32.3%)	67 (17.3%)	95 (24.5%)	
Serie A	83 (29.6%)	71 (25.4%)	69 (24.6%)	57 (20.4%)	

As shown in Table 1, Q1 (27.4%) and Q2 (28.4%) together account for the majority of players, whereas Q3 (22.2%) and Q4 (22.0%) present lower proportions. This trend is consistent when analyzing each league individually: (a) Women's Super League (England) with 55% of players were born in Q1 and Q2; (b) Liga F (Spain) with 58%; (c) Frauen-Bundesliga (Germany) with 53%; (d) Serie A Femminile (Italy) with 55% in; (e) Division 1 (France) with 57%. Notably, Serie A Femminile shows a particularly high representation of Q1 (29.6%), while Liga F stands out with a higher share of Q2 (32.3%). Despite these variations, the association analysis revealed no significant dependency between the league and the birth quartile (*χ*^2^ = 17.808; *p* = .33). Additionally, the association analysis between both variables showed that there was no dependency between the league and the birth quartile.

At the club level, the majority of clubs within each league also exhibited a higher number of first-semester-born players. Specifically: (a) Women's Super League (England): 9 out of 12 clubs had more first-semester players; (b) Liga F (Spain): 14 out of 16 clubs had more first-semester players; (c) Frauen-Bundesliga (Germany): 6 out of 12 clubs had more first-semester players; 5 clubs had more second-semester players, and 1 club had an equal number; (d) Serie A Femminile (Italy): 8 out of 10 clubs had more first-semester players; (e) Division 1 (France): 10 out of 12 clubs had more first-semester players.

**Figure 1:**
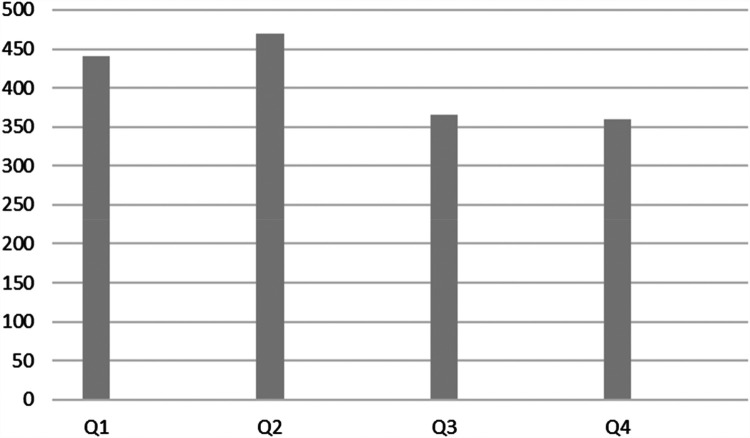
Frequency of players by quarter for all players in the five European leagues.

Figure 1 illustrates the distribution of players' birth dates by quarter across the five European leagues. The figure demonstrates that the first two quarters (Q1 and Q2) account for a larger percentage of players (56%) compared to Q3 and Q4 (44%).

### 3.2 Poisson regression analysis by league

To statistically assess the presence of RAEs, Poisson regression analyses were conducted for the overall sample and for each league individually. The results are presented in Table 2.

**Table 2:** Poisson regression analysis of RAEs by frequency for all players by league.

League	*n*	WB (Mea*n* ± SD)	Tb (Mean ± SD)	*b*₀	*b*₁	ID	D^2^ (McFadden)	*p*-value
Overall (5 Leagues)	1,634	25 ± 15	0.47 ± 0.28	3.65	−0.45	1.57	0.24	<0.001
Women's Super League	297	25 ± 15	0.47 ± 0.28	1.94	−0.43	1.54	0.08	<0.05
Liga F	389	25 ± 15	0.47 ± 0.28	2.19	−0.41	1.50	0.06	<0.05
Frauen-Bundesliga	344	26 ± 15	0.49 ± 0.28	1.95	−0.17	1.19	0.02	0.36
Serie A Femminile	281	24 ± 15	0.45 ± 0.28	1.99	−0.66	1.93	0.16	<0.01
Division 1	323	24 ± 14	0.45 ± 0.26	2.11	−0.64	1.88	0.17	<0.001

WB, week of birth; Tb, Time period of birth; ID, Index of Discrimination [*e*^(–*b*₁)]; *D*^2^: likelihood ratio.

The Poisson regression for the overall sample revealed a significant negative association between time of birth (Tb) and the frequency of players (*b*₁ = −0.45, *p* < 0.001), indicating the presence of RAEs. The Index of Discrimination (ID) was 1.57, suggesting that players born at the beginning of the year are 1.57 times more likely to be selected than those born at the end of the year. The Poisson regression analysis revealed the presence of a significant (*p* < 0.001) overall RAEs in players in the first division of the teams in Division 1 (France). There is also a significant difference (*p* < 0.01) in Serie A Femminile (Italy), and a significant difference (*p* < 0.05) in Liga F (Spain) and Women's Super League (England). There is no significant difference (*p* = 0.36) in Frauen-Bundesliga (Germany). The McFadden's *D*^2^ values indicate a moderate model fit, with higher values in Serie A Femminile (*D*^2^ = 0.16) and Division 1 Féminine (*D*^2^ = 0.17). In contrast, the Frauen-Bundesliga shows the lowest *D*^2^ (0.02), aligning with the non-statistically significant RAEs observed in this league.

**Figure 2:**
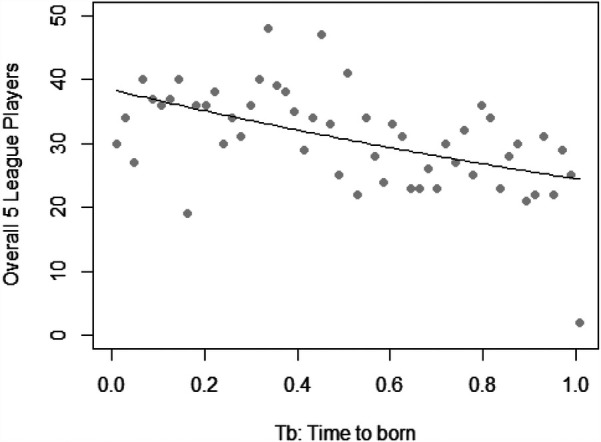
Frequency of week of birth (WB) for all players and poisson regression line for the overall five leagues.

Figure 2 shows the frequency of week of birth and the Poisson regression line for the overall sample. The negative slope of the regression line in Figure 2 indicates a decrease in the number of players born later in the year, consistent with the presence of RAEs.

### 3.3 Distribution of birth dates by playing position

The distribution of players' birth dates according to quartile by playing position is detailed in Table 3. Statistically significant differences were found between the specific position and the birth quartile (*χ*^2^ = 46.77; *p* < .05; ES = .06).

**Table 3:** Association between birth quartile and player position.

Position	Q1 (*n* = 441–27.4%)	Q2 (*n* = 457–28.4%)	Q3 (*n* = 358–22.2%)	Q4 (*n* = 354–22.0%)	*P* [ES]
Goalkeeper	61 (33.3%)	62 (33.9%)	**26** (19.4%)**	34 (20.3%)	*p* < .05 [.06]
Defender	124 (27.4%)	149 (32.9%)	88 (19.4%)	92 (20.3%)	
Midfielder	146 (28.9%)	144 (28.5%)	**126** (25%)*	89 (17.6%)	
Striker	110 (28.0%)	102 (26.0%)	96 (24.4%)	85 (21.6%)	

* More observed than expected values obtained through the z value of the adjusted residual.

** Less observed than expected values obtained through the z value of the adjusted residual.

When analyzing by position, there is a greater presence of players born in the first semester (Q1 and Q2) across all positions, goalkeepers are more frequently born in Q1 (33.3%) and Q2 (33.9%), while their presence is statistically significantly lower in Q3 birth quartile (*Z* < −1.96), indicating an underrepresentation in this quartile Defenders show the highest proportion in Q2 (32.9%, followed by Q1 (27.4%), with Q3 (19.4%) and Q4 (20.3%) being less common. Midfielders are more evenly distributed, but Q3 stands out with a statistically significantly higher representation compared to Q4 (17.6%), the lowest among all quartiles. Strikers follow a more gradual trend, with the highest proportion in Q1 (28.0%) and a continuous decrease toward Q4 (21.6%). When breaking it down by championship, the overall trend shows more players born in the first semester for the entire sample. However, there are two exceptions: forwards in the Frauen-Bundesliga have a higher presence of players born in the second semester, and defenders in England have an equal number of players born in the first and second semesters.

### 3.4 Poisson regression analysis by playing position

Poisson regression analyses were conducted for each playing position, with results summarized in Table 4.

**Table 4:** Poisson regression analysis of RAEs by frequency for all players by playing position.

Position	*n*	WB (Mean ± SD)	Tb (Mean ± SD)	*b*₀	*b*₁	ID	*D*^2^ (McFadden)	*p*-value
Goalkeepers	191	24 ± 15	0.45 ± 0.28	1.64	−0.75	2.11	0.11	<0.01
Defenders	459	26 ± 15	0.49 ± 0.28	2.28	−0.24	1.27	0.03	0.13
Midfielders	522	25 ± 15	0.47 ± 0.28	2.55	−0.54	1.72	0.18	<0.001
Forwards	407	26 ± 15	0.49 ± 0.28	2.20	−0.32	1.38	0.06	0.056

WB, week of birth; Tb, time period of birth; ID, Index of Discrimination [*e*^(–*b*₁)]; *D*^2^: likelihood ratio.

The analyses reveal the presence of significant relative age effects (RAEs) in midfielders (*p* < 0.001, ID = 1.72) and goalkeepers (*p* < 0.01, ID = 2.11). However, no significant RAEs were found among defenders (*p* = 0.13, ID = 1.27), while forwards showed no significant RAEs but presented a marginal trend (*p* = 0.056, ID = 1.38). These findings align with the results of the Poisson regression by demarcation, which identified significant RAEs in midfielders and goalkeepers, but not in defenders or forwards. The McFadden's *D*^2^ values suggest a stronger model fit for goalkeepers (*D*^2^ = 0.11) and midfielders (*D*^2^ = 0.18), supporting the presence of statistically significant relative age effects in these positions. Conversely, defenders (*D*^2^ = 0.03) and forwards (*D*^2^ = 0.06) show lower values, indicating a weaker or non-statistically significant effects.

**Figure 3:**
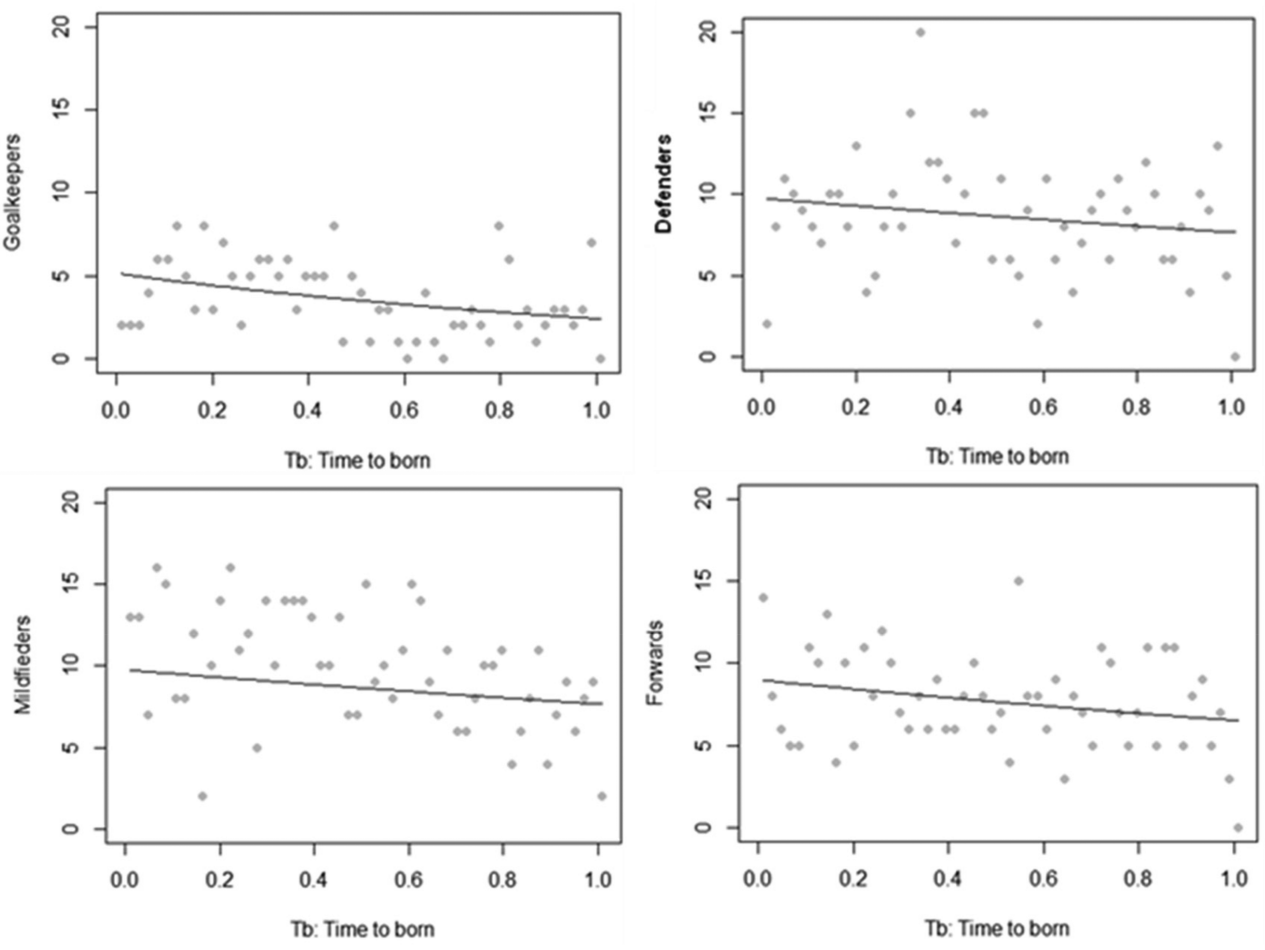
Frequency of week of birth (WB) and poisson regression for each playing position.

Figure 3 exhibits the frequency of week of birth and Poisson regression for each playing position.

### 3.5 Distribution of birth dates by playing position and league

Finally, Table 5 presents the results of the contingency table and association analysis between the variables birth date and playing position for each of the leagues independently. The results obtained indicate that there is no statistically significant association between these variables when analyzed within each league separately, in contrast to the statistically significant association observed when assessed overall.

**Table 5:** Association between birth quartile and player position in top-5 female European leagues.

League	Position	Q1 (*n* = 441–27.4%)	Q2 (*n* = 457–28.4%)	Q3 (*n* = 358–22.2%)	Q4 (*n* = 354–22.0%)	*P* [ES]
Division 1	Goalkeeper	13 (33.3%)	10 (25.6%)	8 (20.5%)	8 (20.5%)	.74 [-]
	Defender	34 (35.1%)	28 (28.9%)	20 (20.6%)	15 (15.5%)	
	Midfielder	22 (22.9%)	30 (31.3%)	27 (28.1%)	17 (17.7%)	
	Striker	24 (26.4%)	24 (26.4%)	23 (25.3%)	20 (22.0%)	
Serie A Femminile	Goalkeeper	11 (30.6%)	10 (27,8%)	5 (13.9%)	10 (27.8%)	.68 [-]
	Defender	24 (28.2%)	23 (27,1%)	19 (22.4%)	19 (22,4%)	
	Midfielder	31 (33.0%)	20 (21.3%)	25 (26.6%)	18 (19.1%)	
	Striker	17 (26.2%)	18 (27.7%)	20 (30.8%)	10 (15.4%)	
Liga F	Goalkeeper	12 (30.0%)	14 (35.0%)	5 (12.5%)	9 (22.5%)	.60 [-]
	Defender	24 (19.2%)	48 (38.4%)	22 (17.6%)	31 (24.8%)	
	Midfielder	34 (29.3%)	36 (31.0%)	20 (17.2%)	26 (22.4%)	
	Striker	30 (28.3%)	27 (25.5%)	20 (18.9%)	29 (27.4%)	
Women's Super Lige	Goalkeeper	13 (36.1%)	12 (33.3%)	2 (5.6%)	9 (25.0%)	.56 [-]
	Defender	21 (25.6%)	20 (24.4%)	21 (25.6%)	20 (24.4%)	
	Midfielder	28 (28.3%)	26 (26.3%)	25 (25.3%)	20 (20.2%)	
	Striker	24 (30.0%)	19 (23.8%)	19 (23.8%)	18 (22.5%)	
Frauen-Bundesliga	Goalkeeper	12 (27.9%)	16 (37.2%)	7 (16.3%)	8 (18.6%)	.35 [-]
	Defender	21 (22.8%)	30 (32.6%)	15 (16.3%)	26 (28.3%)	
	Midfielder	31 (25%)	32 (25.8%)	36 (29%)	25 (20.2%)	
	Striker	15 (22.7%)	14 (21.2%)	19 (28.8%)	18 (27.3%)	

## 4 Discussion

Our results suggest noticeable tendency towards Relative Age Effects in the most important female football leagues in Europe with the exception of German Frauen Bundesliga. This aligns with similar research that analyzed Japan Woman Soccer League (i.e., Nadeshiko League) and stated that RAEs were also present at the top female football league in Japan with a clear predominancy of Q1 born players (19). Similarly, in Spanish female football, the top three divisions, regional, and national teams showed the presence of Relative Age Effects (22). Same results were also found in the Turkish Women's Super League during the 2022–2023 season (24), but it is worth noting that the effects were mild when only the top teams in the league were analyzed, while these effects disappeared in the total sample of the league, possibly due to significant differences in level between the teams analyzed. Curiously, in the case of the Brazilian Female Football First Division (i.e., Campeonato Brasileiro Futebol Feminino A1), Relative Age Effects were trivially present when all players of the competition were analyzed together; however, it was not found when the players were categorized by their specific position (23).

In regards to playing positions and RAEs, our findings indicate significant RAEs among goalkeepers and midfielders, but not among defenders and forwards. This contrasts with findings by Bilgiç and Işin (30) in the 2016, 2018, and 2022 FIFA Women's World Cup. These authors (30) reported statistically significant RAEs across all positions in U17 and among defenders and midfielders in U20. This suggest that age-related selection biases may vary by competition level and tournament structure. Complementarily, Ribeiro et al. (31) observed, in the Women's Football World Cup from 2018 to 2019, a strong overrepresentation of players born in Q1, particularly among midfielders, which aligns with our results, as midfielders exhibited significant RAEs. Moreover, goalkeepers and defenders were found to be influenced by the highest RAEs in Spanish female football national teams and top competitive levels (22), which aligns with our present results, except for the case of defenders in the Women's Super League (England). Additionally, recent research in U.S. youth women's football has reported that RAEs are particularly pronounced among goalkeepers, central defenders, midfielders, and center forwards during the talent identification phase, though these positional biases diminish when players reach the youth national team (40).

A similar study of the top BIG 5 European leagues (i.e., British Premier League, Spanish LaLiga, French Ligue1, Italian Serie A, and German Bundesliga) was conducted by Úbeda-Pastor et al. (41) using a male sample. The results indicated the presence of RAEs in four out of the five leagues (LaLiga, Ligue 1, Serie A, and Bundesliga), with statistically significant overrepresentation of players born in the first quarter of the year. However, no significant differences were found in the English Premier League. Moreover, more recent comparative study has emphasized that while RAEs are robustly evident in male competitions, the magnitude and patterns in female competitions differ (17, 35), suggesting that selection dynamics may operate differently across genders.

On an overall scope, it´s interesting to point out the importance of RAEs in youth stages, since high presence of RAEs in adolescence translates in a presence of RAEs also early adulthood career phase (i.e., younger than 25 years old) in different sports (e.g., rugby, volleyball and basketball) with an special note on football, where results has shown that not only affect early adulthood but also later phases as well (42). In this regard, recent investigations in other sports, such as athletics, have underscored that the developmental trajectories contributing to RAEs are complex and evolve over time, with early advantages potentially diminishing as athletes mature (31, 43).

One explanation for RAE been considered lees influential in female sport could be the lower level of competition among female athletes for positions in elite teams. If an activity is far more popular among boys than girls in a given country, and if similar elite structures exist with a similar selection system, it is not surprising to find higher RAEs among males than among females (17). The second major determinant, physical development, also deserves to be interrogated with regard to potential sex differences. Baxter-Jones (44) suggested that the stronger RAEs among male athletes are the result of the earlier maturation of girls and the higher variance of the maturity status of boys. During the period of selection, there would thus be more significant differences between boys than between girls. Gredin et al. (45) examined psychological risk factors and found that perceptions of sport competence and motivational climate significantly affected athletes’ likelihood of continuing in the sport. Vincent and Glamser (25) argued that social pressures to conform to a socially constructed gender role (i.e., stereotyped definition of femininity) “could make early maturing females less motivated to achieve excellence in competitive sport because of a perception that society does not value female athletic accomplishments in the same way it does those of males”. Thus, early maturing females are more subject to leaving competitive sports than later maturing females (25).

As stated in the introductory part of the present manuscript, female football is an emerging sport in many European countries such as Spain and England (2). A strong growing popularity and competitiveness of the sport often translates into a higher presence of Relative Age Effects at different female football levels, since a higher number of footballers can lead to discrimination against players born in Q3 and Q4 in their selection to participate in different football squads (46). In the case of Luxembourg, due to their limited pool of players, RAEs are not present at any of their either female or male youth football (46). Also, this argument can be extracted from the analysis of Pedersen et al. (21), who studied the historic evolution of RAEs presence at the Women's World Cup U17 and U20, and their findings proved how RAEs weren't present during 2008, 2010, 2012, and 2014 in contrast with more recent editions (2016 and 2018). Even stronger evidence of such effects were detected at the U18 World Cup, where RAEs weren't present over the 2002–2016 period; however, it appeared at the 2018 edition (21). The same tendency as the French Division 1, a league that didn't present RAEs back in 2009 (17) but does recently (e.g., 2023 season) as the present investigation has proved. The Swiss national teams didn't present RAEs either in 2011, attributed to the low number of potential players, but another variable needs to be taken into account: the lack of professionalization (29). This temporal shift underscores the dynamic and evolving nature of RAEs, reflecting changes in youth development and selection policies over time (30). Furthermore, such trends illustrate that as competitive structures mature and professionalization increases, RAEs may emerge or intensify in leagues where they were previously undetectable, as supported by findings from Bezuglov et al. (47), who identified widespread RAEs in European professional soccer, particularly pronounced in more competitive leagues.

The more professionalized the clubs and academies, the stronger RAEs effects in Portuguese female and male football and futsal (48); so definitely, we can say that at the elite level the RAEs level would always be higher, and that aligns with most of our current results in the BIG 5 leagues. Historically speaking, especially at their development level, female football has focused a big share of their resources on the development of female sport adherence, transmission of sport values, and the increase of female participation in football (49). The fact that this stage has been robustly achieved has led to a new stage where teams aim not only at participation and sport values but the competitiveness of their teams as well. In other words, a higher focus on winning translates into a higher level of RAEs as the studies of the higher divisions of female Spanish football showed higher levels of RAEs than the other lower divisions (22, 50).

### 4.1 Limitations, practical implications and future research

Despite the detailed analysis, the study deals with some limitations. The research focused solely on the top-tier leagues during a single season (2023/2024), which may not capture longitudinal trends or account for variations in lower-tier leagues and other countries. Furthermore, factors such as cultural differences, developmental systems, and league-specific regulations were not examined, which could influence the presence and extent of RAEs.

The practical applications of these findings are significant for talent identification and development in women's football. Recognizing the existence of RAEs can help coaches, scouts, and administrators implement strategies to mitigate its impact, such as adjusting scouting practices or providing additional support to later-born players. This could lead to a more equitable selection process, ensuring that talent is recognized regardless of relative age, ultimately enhancing the overall quality of the sport.

Future research should consider longitudinal studies to assess changes in RAEs over multiple seasons and include a broader range of leagues and age groups. Investigating the underlying causes of RAEs in women's football, such as physical maturation rates, social influences, and selection biases, would provide deeper knowledge. Additionally, exploring intervention strategies like bio-banding or alternative age groupings could offer practical solutions to reduce the RAEs' impact on player development and selection.

## Data Availability

The raw data supporting the conclusions of this article will be made available by the authors, without undue reservation.

## References

[1] FIFA. FIFA Women’s World Cup France 2019. Technical Report. Zurich (Switzerland) (2019).

[2] FIFA. Women’s Football: Member Associations Survey Report 2023. Zurich (Switzerland) (2023). Available online at: https://digitalhub.fifa.com/m/28ed34bd888832a8/original/FIFA-Women-s-Football-MA-Survey-Report-2023.pdf (accessed April 4, 2024).

[3] Okholm Kryger K Wang A Mehta R Impellizzeri FM Massey A McCall A. Research on women’s football: a scoping review. Sci Med Football. (2022) 6:549–58. 10.1080/24733938.2020.1868560 36540910

[4] Lago I Lago-Peñas S Lago-Peñas C. Waiting or acting? The gender gap in international football success. Int Rev Sociol Sport. (2022) 57:1139–56. 10.1177/10126902211060727

[5] Cobley S Baker J Wattie N McKenna J. Annual age-grouping and athlete development. Sports Med. (2009) 39:235–56. 10.2165/00007256-200939030-00005 19290678

[6] Smith KL Weir PL Till K Romann M Cobley S. Relative age effects across and within female sport contexts: a systematic review and meta-analysis. Sports Med. (2018) 48:1454–78. 10.1007/s40279-018-0890-8 29536262

[7] Musch J Grondin S. Unequal competition as an impediment to personal development: a review of the relative age effect in sport. Dev Rev. (2001) 21:147–67. 10.1006/drev.2000.0516

[8] Wattie N Cobley S Baker J. Towards a unified understanding of relative age effects. J Sports Sci. (2008) 26:1403–9. 10.1080/02640410802233034 18825541

[9] de la RA Bjørndal CT Sánchez-Molina J Yagüe JM Calvo JL Maroto-Izquierdo S. The relationship between the relative age effect and performance among athletes in world handball championships. PLoS One. (2020) 15:e0230133. 10.1371/journal.pone.0230133 32214322 PMC7098603

[10] Malina RM Cumming SP Rogol AD Coelho-e-Silva MJ Figueiredo AJ Konarski JM Bio-Banding in youth sports: background, concept, and application. Sports Med. (2019) 49:1671–85. 10.1007/s40279-019-01166-x 31429034

[11] Schorer J Cobley S Büsch D Bräutigam H Baker J. Influences of competition level, gender, player nationality, career stage and playing position on relative age effects. Scand J Med Sci Sports. (2009) 19:720–30. 10.1111/j.1600-0838.2008.00838.x 18627551

[12] Smith K Weir P. Late birthday benefits. In: Dixon J Horton S Chittle L Baker J, editors. Relative Age Effects in Sport. New York, NY: Routledge (2020):71–82. 10.4324/9781003030737-7

[13] Gibbs BG Jarvis JA Dufur MJ. The rise of the underdog? The relative age effect reversal among Canadian-born NHL hockey players: a reply to Nolan and Howell. Int Rev Sociol Sport. (2012) 47:644–9. 10.1177/1012690211414343

[14] Morganti, G Kelly AL Apollaro G Pantanella L Esposito M Grossi A Relative age effects and the youth-to-senior transition in Italian soccer: the underdog hypothesis versus knock-on effects of relative age. Sci Med Football. (2023) 7:406–12. 10.1080/24733938.2022.2125170 36103671

[15] Hancock DJ Adler AL Côté J. A proposed theoretical model to explain relative age effects in sport. Eur J Sport Sci. (2013) 13:630–7. 10.1080/17461391.2013.775352 24251740

[16] Wattie N Schorer J Baker J. The relative age effect in sport: a developmental systems model. Sports Med. (2015) 45:83–94. 10.1007/s40279-014-0248-9 25169442

[17] Delorme N Boiché J Raspaud M. The relative age effect in elite sport: the French case. Res Q Exerc Sport. (2009) 80:336–44. 10.1080/02701367.2009.10599568 19650399

[18] Götze M Hoppe MW. Relative age effect in elite German soccer: influence of gender and competition level. Front Psychol. (2021) 11:3725. 10.3389/fpsyg.2020.587023 33542698 PMC7852549

[19] Matsuda S Ishigaki H. Trends in relative age effects of top-level female soccer players: a Japanese study. Percept Mot Skills. (2023) 130:984–98. 10.1177/00315125231162745 36921122

[20] Morales Júnior VR Alves IVG Galatti LR Marques RFR. The relative age effect on Brazilian elite futsal: men and women scenarios. Motriz: Revista de Educação Física. (2018) 23:e101704. 10.1590/s1980-6574201700030016

[21] Pedersen AV Aune TK Dalen T Lorås H. Variations in the relative age effect with age and sex, and over time—elite-level data from international soccer world cups. PLoS One. (2022) 17:e0264813. 10.1371/journal.pone.0264813 35482636 PMC9049515

[22] Sedano S Vaeyens R Redondo JC. The relative age effect in Spanish female soccer players. Influence of the competitive level and a playing position. J Hum Kinet. (2015) 46:129–37. 10.1515/hukin-2015-0041 26240656 PMC4519203

[23] Teoldo I Machado VR Casanova F Cardoso F. Talent map of female soccer: how does the birthplace and birthdate impact the participation of soccer players in Brazilian serie A1 championship? Journal of Human Sport and Exercise. (2023) 18:858–70. 10.14198/jhse.2023.184.10

[24] Bayarslan B Özsoy D Dokuzoğlu G. Relative age effect in turkish women football. The Online Journal of Recreation and Sports. (2023) 12:596–602. 10.22282/tojras.1324079

[25] Vincent J Glamser FD. Gender differences in the relative age effect among US Olympic development program youth soccer players. J Sports Sci. (2006) 24:405–13. 10.1080/02640410500244655 16492604

[26] Barreira J Bueno B Chiminazzo JGC. Relative age effect and age of peak performance: an analysis of women’s football players in the Olympic games (1996–2016). Motriz: Revista de Educação Física. (2021) 27:e1021006921. 10.1590/s1980-65742021006921

[27] Nakata H Sakamoto K. Sex differences in relative age effects among Japanese athletes. Percept Mot Skills. (2012) 115:179–86. 10.2466/10.05.17.PMS.115.4.179-186 23033755

[28] Li Z Mao L Steingröver C Wattie N Baker J Schorer J Relative age effects in elite Chinese soccer players: implications of the ‘one-child’ policy. PLoS One. (2020) 15:e0228611. 10.1371/journal.pone.0228611 32059000 PMC7021294

[29] Romann M Fuchslocher J. Influence of the selection level, age and playing position on relative age effects in Swiss women’s soccer. Talent Development and Excellence. (2011) 3:239–47.

[30] Bilgiç M Işin A. Investigation of relative age effect in female soccer: born to play? Spor Bilimleri Dergisi. (2023) 34:88–97. 10.17644/sbd.1227529

[31] Ribeiro E Barreira J Carraco D Galatti L Götze M Cal Abad CC. The relative age effect in under-17, under-20, and adult elite female soccer players. Science and Medicine in Football. (2024) 8:153. 10.1080/24733938.2022.2164608 36592346

[32] Brustio PR Cobley S Abbott S La Torre A Moisè P Rainoldi A Corrective adjustment procedures as a strategy to remove relative age effects: validation across male and female age-group long jumping. J Sci Med Sport. (2022) 25:678–83. 10.1016/j.jsams.2022.04.007 35644757

[33] Smith KL Koopmann T Weir PL Schorer J. An investigation on main effects and interactions of relative age effects and playing position in female elite football in Germany. Front Sports Act Living. (2024) 6:1459784. 10.3389/fspor.2024.1459784 39421796 PMC11484271

[34] Brustio PR Boccia G Pasquale D Lupo P Ungureanu C N A. Small relative age effect appears in professional female Italian team sports. Int J Environ Res Public Health. (2022) 19(1):385. 10.3390/ijerph19010385 35010643 PMC8750980

[35] Andrew M Finnegan L Datson N Dugdale JH. Men are from quartile one, women are from? Relative age effect in European soccer and the influence of age, success, and playing Status. Children. (2022) 9:1747. 10.3390/children9111747 36421196 PMC9689054

[36] Livefutbol. Livefutbol (2024). Available online at: https://www.livefutbol.com/ (accessed February 10, 2024).

[37] Doyle JR Bottomley PA. Relative age effect in elite soccer: more early-born players, but no better valued, and no paragon clubs or countries. PLoS One. (2018) 13:e0192209. 10.1371/journal.pone.0192209 29420576 PMC5805271

[38] Doyle JR Bottomley PA. The relative age effect in European elite soccer: a practical guide to poisson regression modelling. PLoS One. (2019) 14:e0213988. 10.1371/journal.pone.0213988 30943241 PMC6447143

[39] Cohen AS Kim S-H Wollack JA. An investigation of the likelihood ratio test for detection of differential item functioning. Appl Psychol Meas. (1996) 20:15–26. 10.1177/014662169602000102

[40] Finnegan L van Rijbroek M Oliva-Lozano J Cost R Andrew M. Relative age effect across the talent identification process of youth female soccer players in the United States: influence of birth year, position, biological maturation, and skill level. Biol Sport. (2024) 41:241–51. 10.5114/biolsport.2024.136085 39416506 PMC11475004

[41] Úbeda-Pastor V Guerrero-Jiménez P Llana-Belloch S. Efecto de la edad relativa en cinco ligas europeas de fútbol profesional relative age effect on five European professional soccer leagues. Kronos. (2020) 19:1–7.

[42] Lupo C Boccia G Ungureanu AN Frati R Marocco R Brustio PR. The beginning of senior career in team sport is affected by relative age effect. Front Psychol. (2019) 10:1465. 10.3389/fpsyg.2019.01465 31293489 PMC6606777

[43] Brustio PR Stival M Boccia G. Relative age effect reversal on the junior-to-senior transition in world-class athletics. J Sports Sci. (2023) 41:903–9. 10.1080/02640414.2023.2245647 37555554

[44] Baxter-Jones ADG. Growth and development of young athletes. Sports Med. (1995) 20:59–64. 10.2165/00007256-199520020-00001 7481282

[45] Gredin NV Back J Johnson U Svedberg P Stenling A Solstad BE Exploring psychosocial risk factors for dropout in adolescent female soccer. Sci Med Football. (2022) 6:668–74. 10.1080/24733938.2022.2088843 36540913

[46] Simon C Carson F Faber IR Hülsdünker T. Low prevalence of relative age effects in Luxembourg’s male and female youth football. PLoS One. (2022) 17:e0273019. 10.1371/journal.pone.0273019 35998177 PMC9398004

[47] Bezuglov E Morgans R Butovskiy M Emanov A Shagiakhmetova L Pirmakhanov B The relative age effect is widespread among European adult professional soccer players but does not affect their market value. PLoS One. (2023) 18:e0283390. 10.1371/journal.pone.0283390 36952464 PMC10035832

[48] Figueiredo P Seabra A Brito M Galvão M Brito J. Are soccer and futsal affected by the relative age effect? The Portuguese football association case. Front Psychol. (2021) 12:679476. 10.3389/fpsyg.2021.679476 34122274 PMC8194498

[49] Pfister G. Assessing the sociology of sport: on women and football. Int Rev Sociol Sport. (2015) 50:563–9. 10.1177/1012690214566646

[50] Práxedes A Villar F Del Gil-Arias A Pizarro D Moreno A. Evolution of the relative age effect in Spanish young footballers (U8 to U19). A comparative analysis in elite clubs vs. Low-level clubs. Eur J Hum Mov. (2019) 43:102–14.

